# Supplementary material for: Collaborative intelligence in AI: Evaluating the performance of a council of AIs on the USMLE
Source: PLOS Digit Health. 2025 Oct 9;4(10):e0000787. doi: 10.1371/journal.pdig.0000787 (PMC12510544; doi:10.1371/journal.pdig.0000787)
Supplement: S2 File — (ZIP) [file pdig.0000787.s002.zip › S2 Data Raw data files containing unprocessed question inputs and LLM outputs including transcripts of deliberations/STEP2_Question-97-1_Type-MC-NJ.docx]

**Facilitator** **[Round 1]:** A 57-year-old man is admitted to the hospital 30 minutes after he was found wandering in the streets. Fingerstick blood glucose concentration obtained by paramedics prior to transport was 68 mg/dL. The patient's medical history is unknown. Temperature is 35.8°C (96.5°F), pulse is 80/min, respirations are 16/min, and blood pressure is 106/70 mm Hg. The patient appears disheveled and is not oriented to person, place, or time. Physical examination shows mild conjunctival icterus. Cardiopulmonary examination discloses no abnormalities. Abdominal examination discloses a small fluid wave. There is palmar erythema and 1+ pitting edema of the lower extremities. Gait is broad based and ataxic. The patient is unable to cooperate with ocular or neurologic examinations. ECG shows a regular sinus rhythm and no acute abnormalities. Results of toxicology screening are pending. The most appropriate initial step in management is to administer which of the following? (A) Glucose (B) Vitamin B₁ (thiamine) (C) Vitamin B₂ (riboflavin) (D) Vitamin B₆ (pyridoxine) (E) Vitamin B₁₂ (cyanocobalamin)

**AI_E:** (1) RESTATING THE QUESTION: The presented case is of a 57-year-old man who was found in an distressed state and presented to the hospital. Key findings include confusion, ataxia, conjunctival icterus, fluid wave on abdominal examination indicating likely ascites, palmar erythema, edema of the lower extremities, and broad-based gait. His blood glucose level was measured to be 68 mg/dL, which is at lower end of normal range. The question asks what should be the appropriate initial step in managing this patient. (2) PLANNING: The key assumption in this case is that his symptoms might be a result of alcoholism, malnutrition, or a combination of both due to the presentation of potential liver disease and central nervous system involvement. However, several factors need to be considered - Why is he confused? Is the ataxic gait due to alcohol abuse or a neurological problem? What is the cause of the conjunctival icterus, palmar erythema and pitting edema? These symptoms suggest liver disease, possibly due to alcohol abuse - a common cause of these signs. The vitamin deficiencies could be blamed for some of these symptoms as well. (3) CLINICAL REASONING: Vitamin B1 (thiamine) deficiency could manifest in different ways, one of them being Wernicke's encephalopathy, which would explain the mental confusion and ataxia. In cases of chronic alcoholism, absorption of thiamine is impaired due to damage to cells in the stomach and intestine, leading to an overall deficiency. The liver-related findings - icterus, fluid wave suggesting ascites, palmar erythema and pitting edema - can be markers of advanced liver disease, potentially due to chronic alcohol cirrhosis. (4) CONCLUDING: The best immediate treatment option would be administration of Thiamine, since it can be life-saving in patients showing symptoms of Wernicke's encephalopathy. (5) RESPONSE: Therefore, based on the symptoms of mental confusion, ataxia, and the potential history of alcohol abuse, the initial step in this patient's management is to administer Vitamin B1 (Thiamine), option (B).

**AI_B:** RESTATING THE QUESTION: We are presented with a case of a 57-year-old man who has been found in an unknown medical status and exhibits disorientation, uncooperative behavior, ambulatory abnormalities, and signs of potential liver dysfunction. We are asked to determine the best initial intervention from a range of options, which include providing glucose or several different types of Vitamin B. PLANNING: Assumptions that might be drawn from the initial findings include possibilities of compromised liver function, intoxication, neurological conditions, malnourishment, or some combination. Each assumption impacts the choice of initial intervention. Questioning these, we might ask: Could these symptoms result from B-vitamin deficiency, specifically thiamine (Vitamin B₁), which is essential for neurological functions and energy metabolism and is commonly deficient in malnourished or alcohol-dependent individuals? This alternate assumption reshapes the situation towards a focus on a possible nutritional deficit. CLINICAL REASONING: Let's connect each clinical finding to this alternate assumption. The patient's disorientation, gait abnormalities, and uncooperative behavior may stem from a neurological condition known as Wernicke's encephalopathy, which is often seen in alcoholics and malnourished individuals because of thiamine deficiency. Symptoms such as conjunctival icterus and palmar erythema may result from liver dysfunction, often associated with chronic alcohol use, which can further impact nutritional status. Edema and a fluid wave in the abdomen may suggest cirrhosis, which can also be a signal of long-term alcohol abuse resulting in malnourishment. His blood glucose level, while on the lower end of normal, does not suggest hypoglycemia, moderating the need for glucose administration. CONCLUDING: Seeing the overall picture, the symptoms suggest a neurological condition potentially caused by thiamine deficiency often associated with chronic alcohol misuse, which the patient's physical signs support. Though glucose administration can be an emergency intervention for hypoglycemia, here it might exacerbate thiamine deficiency by increasing its metabolic demands, leading to worsening of neurologic symptoms. Hence, it becomes important to administer thiamine even before sorting out the entire clinical picture. RESPONSE: Thus, the most appropriate initial step in the management of this patient should be to administer Vitamin B₁ (thiamine) (Option B). This can guard against potential aggravation of possible neurological conditions caused by thiamine deficiency, suspected due to the constellation of symptoms in this patient.

**AI_C:** (1) RESTATING THE QUESTION: We're discussing the management of a 57-year-old man, found disoriented and wandering. His blood glucose level was normal when sampled. He presents with confusion, disorientation, mild jaundice as indicated by conjunctival icterus, fluid wave suggesting ascites, palmar erythema, lower limb edema, ataxia, and is uncooperative during neurological or eye exams. We don't know his medical history, nor the results of a toxicology screen, yet. Given these findings, we must decide what substance to administer first: glucose, and various B vitamins (thiamine, riboflavin, pyridoxine, cyanocobalamin). (2) PLANNING: Given the disorientation, ataxia and other symptoms, we might assume alcoholism and its associated malnutrition (hence vitamin deficiencies) as an underlying issue. However, without his history and pending toxicology results, it's merely an assumption. It's wise to consider various B-vitamin deficiencies, but we should question each of those individually. From this reasoning, prioritize which should be administered first. (3) CLINICAL REASONING: Thiamine (B1) deficiency may manifest as Wernicke's encephalopathy (with symptoms like confusion, ataxia, opthalmoplegia) which if left untreated, can develop into Korsakoff syndrome (permanent anterograde/retrograde amnesia). It is typically associated with alcoholism, but can occur in any case of malnutrition. This is a reasonable possibility due to his disorientation and ataxia. Glucose infusion in a thiamine-deficient patient may precipitate or worsen Wernicke’s encephalopathy, so thiamine should be administered before, or together with glucose. Riboflavin (B2), pyridoxine (B6) or cyanocobalamin (B12) deficiencies have neurological features, they differ from what this patient presents. Giving glucose first would be reasonable if the patient was hypoglycemic, but his glucose levels were normal. (4) CONCLUDING: Through our clinical thought process, focusing on the presented symptoms and possible vitamin deficiencies, it seems the most reasonable first step in management would be to administer thiamine, given the patient's presentation suggests a possibility of Wernicke's encephalopathy. (5) RESPONSE: Therefore, the most appropriate initial step in this patient's management is to administer Vitamin B₁ (thiamine) (B).

**AI_D:** (1) RESTATING THE QUESTION: We are presented with a 57-year-old man who was found disoriented in the streets. His blood glucose was relatively normal, and he shows various physical symptoms including conjunctival icterus, abnormal gait, palmar erythema, lower extremity edema, and a fluid wave in his abdomen. Despite elevations in bilirubin levels hinted by icterus, his unknown previous medical history, and inability to cooperate physiologic responses to examinations, we find ourselves tasked with determining the best initial management step. The options presented are all substances which could be administered to the person: glucose (used when hypoglycemia is suspected), thiamine (used in Wernicke's encephalopathy among other conditions), riboflavin, pyridoxine, and cyanocobalamin (B vitamins typically associated with specific nutrient deficiencies). (2) PLANNING: At this point, we are assuming that the patient's current state results from a deficiency or an emerging pathological condition that could be remedied by one of the provided substances. His confusion and disorientation could be due to hypoglycemia, a vitamin deficiency, or perhaps an alcoholic issue (cirrhosis, for instance, given his fluid wave and icterus which can indicate liver dysfunction). Given that glucose levels were normal, we can hypothesize that a vitamin deficiency might be responsible here; however, which specific deficiency might be of concern here? (3) CLINICAL REASONING: The variety of symptoms, including ataxic gate, oculomotor disturbances, confusion, mild jaundice (icterus), and potential signs of ascites (fluid wave) that could be related to liver disease, suggest that this patient might be suffering from Wernicke-Korsakoff syndrome, a condition commonly seen in chronic alcohol abuse, associated with thiamine (Vitamin B1) deficiency. Thiamine is crucial for glucose metabolism in the brain, and its deficiency can lead to neurological symptoms. Furthermore, it's important to note that in a patient susceptible to Wernicke's encephalopathy, administration of glucose before thiamine can precipitate the syndrome. Therefore, it's often recommended to first administer thiamine to such a patient, even before glucose levels are measured. (4) CONCLUDING: Considering the patient's symptoms and the potential implications of glucose administration before correcting a presumed thiamine deficiency, the initial management should aim to resolve the suspected thiamine deficiency. (5) RESPONSE: The most appropriate initial step in management would be to administer Vitamin B1 (Thiamine) (B).

**AI_A:** First, the scenario involves a 57-year-old man who had been found aimlessly wandering the streets and was subsequently hospitalized due to his disoriented state. His blood glucose concentration was recorded to be 68 mg/dL upon preliminary examination by the paramedics, which in the context of his signs and symptoms is not concerning. To clarify a few terms, conjunctival icterus denotes a yellowish discoloration of the conjunctiva, which suggests liver disease. Palmar erythema refers to redness on the palms, which also suggests liver disease. A fluid wave in the abdominal examination signifies ascites or an abnormal buildup of fluid in the abdomen, often linked to cirrhosis. Finally, ataxic gait denotes an unsteady, uncoordinated walk, indicating potential issues in the brain or spinal cord. The primary assumptions based on the scenario are a potential case of chronic liver disease, given the observed symptoms of conjunctival icterus, ascites, palmar erythema and an unstable gait. The disorientation may suggest hepatic encephalopathy, a complication of liver disease where the liver's inability to remove toxins from the blood leads to brain damage. However, other presumptive conditions could be hypoglycemia due to the recorded blood glucose level, but the value is within normal range. We could also consider neurological diseases such as dementia or stroke, but there's no clear evidence of these conditions present. Now, focusing on the primary assumption, a liver disease, and specifically cirrhosis, must be clinically prioritized due to the presence of suggestive physical signs. The disorientation or wandering could be due to hepatic encephalopathy, a neuropsychiatric complication of ow grade liver diseases. There might also be an underlying alcohol use disorder given his disheveled appearance and broad-based, ataxic gait which might denote cerebellar dysfunction, a common finding in chronic alcoholism. Within this context, it's vital to remember that alcoholism can lead to a deficiency in vitamin B1 (thiamine), which can also cause neuropsychiatric disorders. When we examine the management options listed, glucose administration could be useful if the patient was hypoglycemic. However, if it's a liver disease with underlying alcohol use disorder, simply administering glucose without thiamine could precipitate Wernicke encephalopathy, a serious neurologic disorder caused due to thiamine deficiency. Therefore, among the options provided, administering vitamin B1 (thiamine) would be the appropriate primary step. The administration of thiamine before glucose is important because glucose metabolism requires thiamine and therefore its administration in a potentially thiamine-deficient patient can precipitate or exacerbate the aforementioned disorder. Therefore, most appropriate initial step in management is to administer the vitamin B1 (Thiamine), option B. This intervention should precede any glucose administration to reduce the risk of Wernicke encephalopathy, a potentially fatal complication. While further medical management and examination is required for a definitive diagnosis and treatment plan, this primary step is critical given the symptoms and potential assumptions.

**Facilitator** The consensus statement is as follows: The case involves a 57-year-old man found wandering the streets in a disoriented state. His physical examination reveals symptoms such as conjunctival icterus, palmar erythema, a fluid wave in the abdomen, and an ataxic gait, which suggest potential liver disease, possibly cirrhosis. His disorientation could be indicative of hepatic encephalopathy, a neuropsychiatric complication of liver disease. The patient's disheveled appearance and broad-based, ataxic gait might also suggest an underlying alcohol use disorder, which can lead to vitamin B1 (thiamine) deficiency and associated neuropsychiatric disorders. Given the patient's symptoms and the potential implications of glucose administration before correcting a presumed thiamine deficiency, the initial management should aim to resolve the suspected thiamine deficiency. Therefore, the most appropriate initial step in management is to administer Vitamin B1 (Thiamine). This intervention should precede any glucose administration to reduce the risk of Wernicke encephalopathy, a potentially fatal complication. While further medical management and examination is required for a definitive diagnosis and treatment plan, this primary step is critical given the symptoms and potential assumptions.
